# A quantitative comparison of two measures of postpartum depression

**DOI:** 10.1186/s12888-022-03836-z

**Published:** 2022-03-19

**Authors:** Ditte-Marie Leegaard Holm, Jan Wohlfahrt, Marie-Louise Hee Rasmussen, Giulia Corn, Mads Melbye

**Affiliations:** 1 Department of Epidemiology Research, Statens Serum Institut, Artillerivej 5, 2300 Copenhagen, Denmark; 2 Department of Clinical Medicine, University of Copenhagen, Copenhagen, Denmark; 3 Department of Genetics, Stanford University School of Medicine, Stanford, CA USA; 4 Center for Fertility and Health, Norwegian Institute of Public Health, Oslo, Norway; 5 K.G. Jebsen Center for Genetic Epidemiology, Norwegian University of Science and Technology, Trondheim, Norway

**Keywords:** Epidemiology, Psychiatric status rating scales, Postpartum, Depression

## Abstract

**Background:**

Studies investigating the prevalence and risk factors for postpartum depression (PPD) have used different definitions. Some studies have used a high score on the Edinburgh Postnatal Depression Scale (EPDS) to define PPD, whereas others have used information on antidepressant medication use and/or diagnostic information on treatment for depression at a psychiatric hospital. We wanted to compare results using these two approaches to evaluate to what degree results can be compared. Moreover we wanted to evaluate, whether use of EPDS or PPAT (defined below) leads to identification of different risk factor profiles.

**Methods:**

We identified women who delivered a child between 1 January 2014 and 31 December 2016 in Copenhagen or in one of the municipalities that were part of the Danish Health Visitors’ Child Health Database. The potential risk factors were demographic factors and pregnancy- and obstetrical events. Outcomes of interest were an EPDS score ≥ 13, use of antidepressants (ATC: N06A) and/or a diagnosis of depression (F32) within six months after birth. Use of antidepressants and/or diagnosis of depression will be referred to as postpartum antidepressant treatment (PPAT). Agreement between EPDS ≥ 13 and PPAT was evaluated by the kappa coefficient. Associations between risk factors and the two outcomes (EPDS ≥ 13 and PPAT) were estimated by risk ratios (RR) using log-linear binomial regression. Presence of a systematic difference between RRs based on EPDS ≥ 13 (RR_EPDS≥13_) and PPAT (RR_PPAT_) was evaluated in a meta-regression approach weighted by inverse-variance and with logarithm of the RRs as outcome.

**Results:**

The estimated PPD prevalence using EPDS ≥ 13 was 3.2% and of PPAT 0.4%. The agreement between the two measures was small (Kappa = 0.08), but their risk factor profile was very similar with no systematic difference between them.

**Conclusions:**

Using the two different methods of case identification produced different prevalence estimates, but a similar risk factor profile. The differences in estimated prevalence and low agreement suggest that the two measures identify different potential PPD cases and using only one of the methods in defining PPD would underestimate PPD prevalence. The similar risk factor profile suggests that the considered risk factors are involved in the general development of PPD.

**Supplementary Information:**

The online version contains supplementary material available at 10.1186/s12888-022-03836-z.

## Background

Postpartum depression (PPD) is defined as a non-psychotic episode of depression that begins in or extends into the postpartum period [1]. Up to around 17% of all mothers experience PPD depending on the assessment criteria used [2, 3]. It is debated whether different phenotypes of peri- and postpartum depression exist [4], and moreover whether PPD is a distinct disorder or similar to depression occurring outside the postpartum period [5]. Onset of timing is also highly debated. In International Classification of Diseases 10^th^ revision (ICD-10) symptoms have to onset within 6 weeks of delivery [6], whereas in Diagnostic and Statistical Manual of Mental Disorders 5^th^ edition (DSM-V) symptoms have to onset during pregnancy or within 4 weeks following delivery [7]. However, in previous research onset of symptoms range between peripartum and up to 12 months postpartum [4, 8–10]. Thus, even though many researchers have studied PPD, there are still many factors that are unknown and discussed.

Healthcare workers can identify potential cases with help from the Edinburgh Postnatal Depression Scale (EPDS), the Patient Health Questionnaire (PHQ-9), Postnatal Depression Screening Scale (PDSS), and Bromley Postnatal Depression Scale (BPDS) among others [11–13]. Various tools have previously been used in research to identify cases due to different study designs. For instance when using surveys in clinical studies, EPDS has been commonly used to identify PPD cases [14–17], however, various cut-off scores have been used [14, 15, 17, 18], and in studies with administrative data linkage information of prescriptions of antidepressants and/or psychiatric inpatient or outpatient treatment for depression after birth (from now on referred to as “postpartum antidepressant treatment”) (PPAT) has been commonly used [19–21].

PPD is a serious mental disease that not only impacts the women themselves, but also mother-infant bonding, and cognitive and emotional problems of the child have been described [22]. Furthermore, research has shown that the recurrence of PPD is high [21]. As of now, a great effort has been put into identifying risk factors for PPD. Commonly reported risk factors are: a psychiatric history, and perinatal symptoms such as depression and anxiety during pregnancy [4, 9, 18, 23, 24]. Moreover stressful life events, lack of social support, and marital conflicts have been reported as possible risk factors [23, 25]. Also obstetrical factors, such as preeclampsia, gestational diabetes, fetal stress, preterm birth, low birth weight, caesarean section, hyperemesis gravidarum, and postpartum haemorrhage, have been investigated as possible risk factors for PPD with inconsistent results [9, 10, 14, 16, 26]. However, even though many studies have tried to identify possible risk factors of PPD, it remains unknown whether use of EPDS or PPAT to identify PPD cases leads to identification of a different risk factor profile.

### Aims of the study

The overall aim of this study was to investigate to what degree results based on Edinburgh Postnatal Depression Scale or PPAT can be compared. Firstly, we compared the prevalence of PPD based on the two measures and the agreement between the two measures. Secondly, we investigated whether their estimated risk factor profile differed.

## Methods

### Study population

We identified women with a registered live-birth in the Danish Medical Birth Registry (MBR) between 1 January 2014 and 31 December 2016 in either Copenhagen or in one of the municipalities that took part in the Danish Health Visitors’ Child Health Database (DHVCHD).

(see “Standardised measuring the health of infants and toddlers in community health services” [27] among others [28, 29] for more details).

In the main analysis, we excluded women with any registered mental illness (ICD-8: 29, 30 or ICD-10: F0-F9) in the National Patient Registry (NPR) or Psychiatric Central Research Register (PCRR). We also excluded women, with a registration of psychoanaleptics (ATC: N06) in the Danish National Prescription Register (DNPR) prior to birth. In a supplementary analysis we did not impose these restrictions in order to evaluate if our prevalences were comparable with previous studies.

### Covariates

We identified potential risk factors by using information from the Danish Civil Registration System (CRS), the MBR, the NPR and Statistics Denmark. The registries used for this study are described in detail in “Supplementary”. Demographic factors included: maternal age at birth, parity, and highest attained educational level at birth. We also identified pregnancy-related and obstetrical events registered between conception and two weeks postpartum. The identified variables were (for ICD-10 diagnoses see “Supplementary”): birth weight (< 2500 g and ≥ 2500 g only singletons), gestational age (< 37 weeks and ≥ 37 weeks), twins, method of delivery (vaginal and caesarean section), gestational hypertension, preeclampsia, threatened abortion, hyperemesis gravidarum, gestational diabetes, maternal care for known or suspected fetal problems, labour and delivery complicated by fetal stress (distress), perineal laceration during delivery, postpartum haemorrhage, and puerperal sepsis.

### Outcomes

Outcome of interest was PPD defined by two different measures, the EPDS and PPAT. We classified an EPDS score ≥ 13 as being a case of PPD based on previous literature [14, 16, 17, 30]. By choosing a cut-point that is frequently used in the literature, we facilitate a comparison of studies that use EPDS and PPAT. As part of the general healthcare system in Denmark, all new mothers are offered visits by a healthcare professional postpartum, and usually at the 6–8 week visit the mothers are screened with the EPDS for PPD.

For the analyses involving the EPDS we excluded the following women: if they had not filled in the EPDS questionnaire within 6 months after giving birth, if they had not answered all 10 questions, and if the registered total score did not correspond to the sum of the single questions. In case a woman had completed more than one questionnaire per birth, we used the one with the highest EPDS score. If only the registered total score was available and no information on the single questions was provided, we considered the total score to be valid. In the dataset for Copenhagen we had the answers to all 10 questions of the EPDS, and we therefore chose to exclude women that had not answered all the questions. In DHVCHD, we sometimes only had the total score, and therefore we considered the total score to be valid.

The second measure was a depression diagnosis (ICD-10 = F32) registered in the NPR or a registration of prescribed antidepressant medications (ATC = N06A) in the DNPR within six months after birth. Only women referred to the hospital can get an ICD-10 diagnosis. The second measure was based on nationwide registers, thus in the municipalities where EPDS was known, PPAT was also known. Some previous studies on PPD have also included women diagnosed with F53.0. However, for the purpose of this paper, we decided not to do this because this group is rather heterogeneous, and we did not think that the potential benefit of the increase in sensitivity outweighed the drawbacks of the decrease in positive predictive value by including this group.

### Statistical analyses

Agreement between EPDS ≥ 13 and PPAT was evaluated by the kappa coefficient.

Associations between risk factors and the two outcomes (EPDS ≥ 13 and PPAT) were estimated by risk ratios (RR) using log-linear binomial regression with adjustment for maternal age (≤ 24, 25–29, 30–34, and ≥ 35), parity (1, 2, and ≥ 3), and highest attained education (≤ 13 years of schooling, 14–16 years of schooling, and ≥ 17 years of schooling). We adjusted for the same factors for both outcomes and for all risk factors in order to facilitate the comparisons of the estimated risk ratios.

As the datasets for Copenhagen and DHVCHD were placed on different servers, we estimated the logRR’s, separately within the two cohorts and then weighted them together using inverse-variance-weighting. The exponential function of the weighted estimates is presented in the figures and tables. Missing information was handled differently depending on whether the risk factor was an adjusting variable or the exposure of interest; in the first case we used mode imputation, while in the second births with missing information were excluded from the analysis. Only results from the pooled analysis are presented.

Presence of a systematic difference between RRs based on EPDS ≥ 13 (RR_EPDS≥13_) and PPAT (RR_PPAT_) was evaluated in a meta-regression approach weighted by inverse-variance and with logarithm of the RRs as outcome. The regression included a categorical variable with a level for each risk factor and a binary variable indicating whether the estimate was based on EPDS ≥ 13 or PPAT. The *p*-value for the Wald test for the latter variable was used to evaluate whether there was a systematic difference between the two measures.

In order to compare our prevalence of PPD with other studies in the literature that did not restrict on prior psychiatric history but included women with psychiatric history, we made an additional analysis including these women.

All the statistical analyses were performed in SAS version 9.4 for Windows.

## Results

The study was based on two cohorts. In the Copenhagen cohort there were 19,795 women with 20,738 births between 1 January 2014 to 31 December 2016, and in the DHVCHD cohort, there were 35,032 women with 36,501 births in the same period (Table 1). In the two cohorts combined the prevalence of EPDS ≥ 13 was 3.2% and the prevalence of PPAT was 0.4%.

**Table 1:** Distribution of demographic factors and pregnancy-related and obstetrical events

	**Total number of women giving births**	**Number of women with available EPDS scores N (%)**
**Total**	57,239	34,415 (60.1%)
**Cohort**		
Copenhagen	20,738	12,676 (61.1%)
DHVCHD	36,501	21,739 (59.6%)
**Maternal age**		
≤ 24	4803	2881 (60.0%)
25–29	17,646	11,268 (63.9%)
30–34	21,232	12,455 (58.7%)
≥ 35	13,558	7811 (57.6%)
**Parity**		
1	29,957	21,250 (70.9%)
2	19,413	9662 (49.8%)
≥ 3	7468	3333 (44.6%)
Missing parity^a^	401	170 (42.4%)
**Highest attained education**		
≤ 13 years of schooling	17,499	10,475 (59.9%)
14–16 years of schooling	18,427	11,779 (63.9%)
≥ 17 years of schooling	16,192	10,354 (63.9%)
Missing maternal education^b^	5121	1807 (35.3%)
**Preterm**		
No	53,939	32,672 (60.6%)
Yes	2723	1531 (56.2%)
Missing gestational age^c^	577	212 (36.7%)
**Low birth weight**		
No	53,649	32,651 (60.9%)
Yes	1758	1016 (57.8%)
LBW – twins^c^	967	376 (38.9%)
Missing birth weight^c^	865	372(43.0%)
**C-section**		
No	45,922	27,848 (60.6%)
Yes	10,749	6362 (59.2%)
Missing birth method^c^	568	205 (36.1%)
**Twins**		
No	56,272	34,039 (60.5%)
Yes	967	376 (38.9%)
**Gestational hypertension**		
No	56,039	33,644 (63.0%)
Yes	1200	771 (64.3%)
**Preeclampsia**		
No	55,612	33,367 (60.0%)
Yes	1627	1048 (64.4%)
**Threatened abortion**		
No	56,169	33,777 (60.1%)
Yes	1070	638 (59.6%)
**Hyperemesis gravidarum**		
No	56,343	33,908 (60.2%)
Yes	896	507 (56.6%)
**Gestational diabetes**		
No	55,548	33,514 (60.3%)
Yes	1691	901 (53.3%)
**Maternal care for known or suspected fetal problem**		
No	52,414	31,697 (60.5%)
Yes	4825	2718 (56.3%)
**Fetal stress**		
No	45,833	27,072 (59.1%)
Yes	11,406	7343 (64.4%)
**Perineal laceration**		
No	29,411	16,691 (56.8%)
Yes	27,828	17,724 (63.7%)
**Postpartum haemorrhage**		
No	43,945	26,313 (59.9%)
Yes	13,294	8102 (60.9%)
**Puerperal sepsis**		
No	56,904	34,217 (60.1%)
Yes	335	198 (59.1%)

^a^When adjusting for parity the observations with missing information were imputed to 1. They were excluded when parity was the exposure of interest

^b^When adjusting for highest attained education the observations with missing information were imputed to “14–16 years of schooling.” They were excluded when highest attained education was the exposure of interest

^c^These observations were excluded when looking at preterm (respectively low birth weight or birth method) as exposure of interest

### Agreement between a high EPDS score and PPAT

Table 2 shows the agreement between the two measures. Among births in women with EPDS ≥ 13, 4.5% had PPAT, and among births in women with PPAT, 44.6% had EPDS ≥ 13. Accordingly, the agreement between the two measures was small (Kappa = 0.08).

**Table 2:** Agreement between EPDS ≥ 13 and PPAT

		**PPAT**		
		**No (n)**	**Yes (n)**	**% PPAT: Yes**
**EPDS ≥ 13**	**No (n)**	33,244	62	0.19%
	**Yes (n)**	1059	50	4.51%
	**% EPDS ≥ 13: Yes**	3.09%	44.64%	

### Comparison of risk factor profile for a high EPDS score and PPAT

As the focus of this study was to assess a possible systematic difference in the risk factor profile for the two measures, the specific RRs for each risk factor is only presented in the “Supplementary” (demographic variables, pregnancy-related and obstetrical events: Table A1 and A2) and not further described here.

**Fig. 1:**
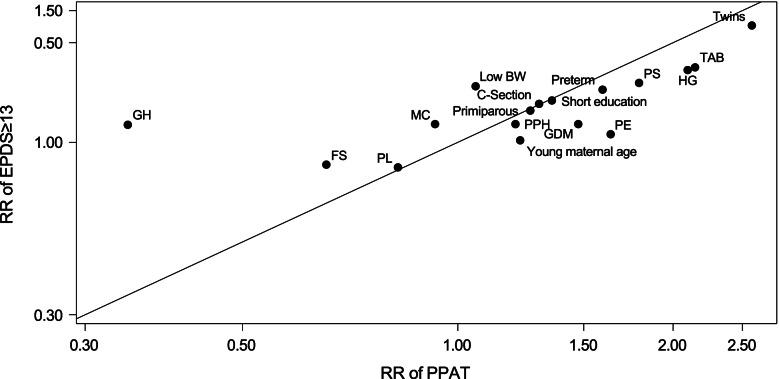
Association between the RR of PPAT and the RR of EPDS ≥ 13 for demographic factors and pregnancy-related and obstetrical events. Based on adjusted RR’s. We adjusted for maternal age, parity, and highest attained education. If the two measures have exactly the same risk factor profile, the points would be on the solid line. Young age: Maternal age ≤ 24 years, Primiparous: Parity 1, Short education: ≤ 13 years of schooling, C-section, FS: Fetal stress, GH: Gestational hypertension, GDM: Gestational diabetes HG: Hyperemesis gravidarum, Low BW: Low birth weight, MC: Maternal care for known or suspected fetal problems, PE: Preeclampsia, PL: Perineal laceration, PPH: Postpartum haemorrhage, Preterm, PS: Puerperal sepsis, TAB: Threatened abortion, and Twins

Figure 1 shows the association between the RR of PPAT (RR_PPAT_) and the RR of EPDS ≥ 13 (RR_EPDS≥13_) according to demographic factors and pregnancy-related and obstetrical events after adjustment for demographic factors. The variables appeared to have a similar association with EPDS ≥ 13 and with PPAT. Thus, we found no significant systematic difference between the two adjusted RR’s (*p* = 0.84). Figure A in the appendix shows a similar association between the RR of PPAT (RR_PPAT_) and the RR of EPDS ≥ 13 (RR_EPDS≥13_) as outcome definition and based on crude RR’s.

When not restricting to women without psychiatric history the prevalence of EPDS ≥ 13 was 4.3% and PPAT 2.7%, respectively.

## Discussion

The aim of the present study was to investigate to what extent results based on EPDS or PPAT can be compared. We found the prevalence of PPD to be 3.2% when using the EPDS ≥ 13 criteria, and 0.4% when using PPAT. In addition, we found low agreement between the two measures. However, when evaluating risk factor profiles for PPD, the two definitions gave similar results, concerning demographic factors and pregnancy-related and obstetrical events.

Our prevalence figures and the fact that we found of a higher prevalence of PPD when using EPDS compared to PPAT is compatible with the previous literature. Thus, in a Danish study of PPD that also excluded prior psychiatric history, the incidence proportion for use of antidepressants and antipsychotics was 7.72 per 1000 birth (0.77%) the first three months postpartum [8].

Studies based on the EPDS, have tended not to restrict on prior psychiatric history. When we likewise in additional analyses included psychiatric history, our estimated prevalence of EPDS ≥ 13 was 4.3%, which is comparable with other European studies that estimated the prevalence to be 4.0%-6.9% [14, 16, 31]. The large difference in prevalence according to the measure of definition used most likely reflects that EPDS is a screening method [30] and underline the importance of taking the used measure into consideration when comparing studies.

Furthermore, we found a low agreement between PPD cases based on EPDS ≥ 13 compared to on PPAT. To our knowledge no other studies have investigated the agreement between these two measures. We found that only 4.5% of the women that had a high score on the EPDS were also registered with PPD based on PPAT. This could be due to EPDS only being a screening method, and a high score is not sufficient to diagnose PPD. A clinical interview is needed in order to evaluate, whether the mother is suffering of depression or not, and perhaps the health care worker considered the symptoms as transient. In other words, perhaps EPDS captures cases with mild symptoms, and PPAT captures cases with moderate-to-severe symptoms. Moreover both the women and doctors could be restrictive towards antidepressants if the women were breastfeeding, since not all antidepressants are recommended when breastfeeding and others not thoroughly investigated [32]. Furthermore, the healthcare professionals screen for PPD at the 6–8 weeks visit, however, it might take some time before the women are prescribed antidepressants or are referred to the hospital, and therefore the time window for the PPAT was defined as 6 months. However, this might on the other hand also have contributed to the EPDS-PPAT discordance, if the women were clinically well when they filled in the EPDS, but later developed a depression. Only 44.6% that were treated with PPAT also had a high score on the EPDS. One explanation for this could be that the women were diagnosed with another psychiatric disease than depression after birth (e.g. an anxiety disorder) that required treatment with antidepressants. Furthermore a Danish study estimated the incidence for first-time use of antidepressants and antipsychotics in the postpartum period to be most frequent in the first month postpartum (3.81 per 1000 births) [8]. Thus, some patients may have been treated for depression shortly after they gave birth and before they completed the questionnaire. This situation would most likely result in a lower score on the EPDS. Finally, a cut-off score of ≥ 13 for a diagnosis of PPD could have been too high (due to the sensitivity of EPDS [30]), however, those with a lower EPDS are less likely to be identified with PPAT.

Overall, the results suggest that none of the two measures identify all potential cases of PPD, and by using just one of them, not all PPD cases will most likely be detected.

We found a similar risk factor profile for PPD using both the definition based on EPDS ≥ 13 and PPAT. Therefore, we did not find evidence supporting that one measure should be preferred to the other when studying risk factors for PPD. Although the risk factor profile for the two measures were similar, the identified PPD cases was different. This suggests that the demographic factors and pregnancy-related and obstetrical events, we addressed in this study, are involved in the development of PPD in general.

Several aspects of our study strengthened our comparison between the EPDS and PPAT measures of PPD. For most women we had information regarding both EPDS and PPAT, which enabled us to perform a direct comparison of the two measures. By using the unique Danish registers that cover the entire population of Denmark, we had mandatorily reported information regarding demographic variables and pregnancy-related and obstetrical event. All information was reported prior to the diagnosis of PPD, which limited the chance for differential misclassification of the information. Furthermore, the rich data material allowed us to evaluate potential differences for many different risk factors. Finally, a previous study found that different subtypes of PPD exist based on e.g. previous psychiatric history [15]. Thus, to achieve a more homogenous study group, the main focus of this study was women without a psychiatric history.

Limitations include rather low prevalence of some of the risk factors, which induced low statistical power, making it difficult to identify and discuss specific risk factors. However, we have enough statistical power to evaluate the overall association between the two measures, and that was the main focus of the study. We do not know how many PPD cases, general practitioners refer to psychiatrists/psychologists in private practice, and we therefore do not know how common it is. That may account for some of the low agreement between the two measures. The use of psychiatric diagnoses and ATC numbers to define PPD sometimes differ between studies, which can make comparison difficult between studies. In Denmark, ethnicity is homogenous, and our results are therefore perhaps not generalizable to societies that are more heterogeneous. It would therefore be interesting to reproduce the study in a sample with a different demographics profile.

In conclusion, we found a low agreement between the symptom-based and the treatment-based measures of PPD. However, the risk factor profiles were rather similar between the two measurements suggesting that most of the demographic factors and pregnancy-related and obstetrical events, we investigated for, were associated with the development of PPD in general rather than linked to special subgroups of PPD with noticeable symptoms or larger need for treatment. Thus, both measures are useful when investigating risk factors for PPD.

## Supplementary Information

**Additional file 1.**

## Data Availability

The data does not belong to us, but can become available for research upon reasonable request by submitting a research protocol to the Danish Data Protection Agency. Once Data Protection Agency permission has been received, data can be applied for at the Ministry of Health’s Research Service (Forskerservice) at forskerservice@ssi.dk. If interested in the Danish Health Visitors’ Child Health database an application form can be filed in (Statens Institut for Folkesundhed: www.sdu.dk/sif/-/media/images/sif/projekter/projekter_dokumenter/databasen_boerns_sundhed/2020/skabelon+-+ansoegning+om+data.docx, and mailed to the Executive Committee of the Danish Health Vistors’ Child Health Database.

## Abbreviations

PPD: Postpartum depression
EPDS: Edinburgh Postnatal Depression Scale
PPAT: Postpartum antidepressant treatment
RR: Risk ratios
MBR: The Danish Medical Birth Registry
DHVCHD: The Danish Health Visitors’ Child Health Database
ICD-8 and ICD-10: International Classification of Diseases 8^th^ and 10^th^ revision
ATC: Anatomical Therapeutic Chemical Classification System
NPR: The National Patient Registry
PCRR: Psychiatric Central Research Register
DNPR: The Danish National Prescription Register
CRS: The Danish Civil Registration System
C-section: Caesarean section
FS: Fetal stress
GH: Gestational hypertension
GDM: Gestational diabetes
HG: Hyperemesis gravidarum
Low BW: Low birth weight
MC: Maternal care for know or suspected fetal problems
PE: Preeclampsia
PL: Perineal laceration
PPH: Postpartum haemorrhage
PS: Puerperal sepsis
TAB: Threatened abortion
