# Supplementary material for: A quantitative comparison of two measures of postpartum depression
Source: BMC Psychiatry. 2022 Mar 19;22:202. doi: 10.1186/s12888-022-03836-z (PMC8933929; doi:10.1186/s12888-022-03836-z)

SUPPLEMENTARY INFORMATION

Data sources

The Danish Civil Registration System (CRS) was established in 1968 and holds information such as demographic factors. All Danish residents are assigned a unique identification number at birth that enables linkage with Denmark's different national registries (1).

The Danish Medical Birth Registry (MBR) was established in 1968 and has been computerized since 1973. It holds data on all live-births and stillbirths in Denmark, if the mother has a permanent residence in Denmark. From this registry, researchers can gain information about gestational age, birth weight, sex, apgar score etc. (2).

The National Patient Registry (NPR) holds information on all hospitalizations in somatic wards from 1977, and since 1995 also all outpatient activities, and activities in psychiatric wards. Since 1994 all diagnoses are recorded using the International Classification of Diseases 10-edition, and from 1977-1993 ICD-8 was used (3).

The Psychiatric Central Research Register (PCRR) holds information on hospitalizations in psychiatric wards since 1969 and is considered complete from 1970. From 1995, it has also recorded information about outpatient treatment and emergency room contacts. The diagnoses are either coded with ICD-8 (1977-1993) or ICD-10 (1994-)(4).

The Danish National Prescription Register (DNPR) holds information on prescription drugs sold in Denmark since 1994 – it is considered complete from 1995. From this registry

researchers can gain information regarding date of dispensing, product name, Anatomical Therapeutic Chemical Classification (ATC) code, and number of dose units in the package (5).

The Danish Health Visitors' Child Health Database (DHVCHD) holds information on children born since 2002. The healthcare visitors also register the mothers' emotional state in the postpartum period. In 2016, 34 municipalities in Denmark were part of DHVCHD(6).

In Copenhagen, data were collected in a separate database.

Definition of pregnancy-related and obstetrical events

Pregnancy related and obstetrical events were identified in the period between conception and two weeks postpartum in the NPR using the ICD-10 diagnoses reported in the following table.

**ICD-10 diagnoses**	**Definition**
O13	Gestational hypertension
O14-O15	Preeclampsia
O20	Threatened abortion
O21	Hyperemesis gravidarum
O24.4	Gestational diabetes
O35-O36	Maternal care for known or suspected fetal problems
O68	Labour and delivery complicated by fetal stress (distress)
O70	Perineal laceration during delivery
O72 (+ additional code of bleeding > 500 mL)	Postpartum haemorrhage
O85	Puerperal sepsis
O80, O81, O83.0-O83.2 O83.7, O83.8A, O84.0, O84.1 O84.7, and O84.8A	Vaginal Birth
O82.0, O82.1, O84.2, and O84.3	Caesarean section

References

1. Pedersen CB. The Danish Civil Registration System. Scandinavian journal of public health. 2011;39(7 Suppl):22-5.

2. Knudsen LB, Olsen J. The Danish Medical Birth Registry. Danish medical bulletin. 1998;45(3):320-3.

3. Lynge E, Sandegaard JL, Rebolj M. The Danish National Patient Register. Scandinavian journal of public health. 2011;39(7 Suppl):30-3.

4. Mors O, Perto GP, Mortensen PB. The Danish Psychiatric Central Research Register. Scandinavian journal of public health. 2011;39(7 Suppl):54-7.

5. Kildemoes HW, Sorensen HT, Hallas J. The Danish National Prescription Registry. Scandinavian journal of public health. 2011;39(7 Suppl):38-41.

6. Databasen Børns Sundhed PSPT. Sundhedsprofil for børn født i 2016 fra Databasen Børns Sundhed. 2018.

Table A1. The RR of EPDS≥13 according to the demographic factors, pregnancy-related and obstetrical events

	No of births with available EPDS score	Of which with EPDS≥13	RR (CI 95 %)	aRR (CI 95%)[1]
**Maternal age**				
≤ 24	2,881	108	1.23 (1.01-1.49)	1.01 (0.82-1.25)
≥ 25	31,534	1,001	1 (reference)	1 (reference)
**Parity**				
1	21,250	742	1.23 (1.09-1.39)	1.25 (1.09-1.42)
≥2	12,995	364	1 (reference)	1 (reference)
**Highest attained education**				
≤ 13 years of schooling	10,475	388	1.35 (1.19-1.53)	1.34 (1.16-1.53)
>13 years of schooling	22,133	643	1 (reference)	1 (reference)
**Preterm[2]**				
No	32,672	1,032	1 (reference)	1 (reference)
Yes	1,531	72	1.49 (1.18-1.88)	1.44 (1.14-1.83)
**Low birth weight (singletons)**				
No	32,651	1,024	1 (reference)	1 (reference)
Yes	1,016	48	1.54 (1.16-2.04)	1.48 (1.11-1.96)
**C-section**				
No	27,848	849	1 (reference)	1 (reference)
Yes	6,362	255	1.32 (1.15-1.52)	1.31 (1.14-1.50)
**Twins**				
No	34,039	1,083	1 (reference)	1 (reference)
Yes	376	26	2.25 (1.55-3.27)	2.25 (1.55-3.28)
**Gestational hypertension**				
No	33,644	1,080	1 (reference)	1 (reference)
Yes	771	29	1.16 (0.81-1.67)	1.13 (0.79-1.63)
**Preeclampsia**				
No	33,367	1,072	1 (reference)	1 (reference)
Yes	1048	37	1.12 (0.81-1.54)	1.06 (0.76-1.46)
**Threatened abortion**				
No	33,777	1,075	1 (reference)	1 (reference)
Yes	638	34	1.72 (1.23-2.40)	1.69 (1.21-2.35)
**Hyperemesis gravidarum**				
No	33,908	1,082	1 (reference)	1 (reference)
Yes	507	27	1.70 (1.17-2.47)	1.65 (1.14-2.40)
**Gestational diabetes**				
No	33,514	1,077	1 (reference)	1 (reference)
Yes	901	32	1.16 (0.82-1.64)	1.14 (0.80-1.61)
**Maternal care for known or suspected fetal problem**				
No	31,697	1,009	1 (reference)	1 (reference)
Yes	2,718	100	1.14 (0.93-1.40)	1.14 (0.93-1.39)
**Fetal stress**				
No	27,072	890	1 (reference)	1 (reference)
Yes	7,343	219	0.90 (0.78-1.05)	0.85 (0.74-0.99)
**Perineal laceration**				
No	16,691	578	1 (reference)	1 (reference)
Yes	17,724	531	0.86 (0.77-0.97)	0.84 (0.75-0.94)
**Postpartum haemorrhage**				
No	26,313	818	1 (reference)	1 (reference)
Yes	8,102	291	1.16 (1.01-1.33)	1.14 (1.00-1.30)
**Puerperal sepsis**				
No	34,217	>1,097[3]	1 (reference)	1 (reference)
Yes	198	<12[3]	1.54 (0.81-2.92)	1.51 (0.80-2.87)

[1]Adjusted for maternal age, parity, and highest attained education.
[2] We repeated the analysis including only singletons and found RR=1.40 (1.09-1.81) and aRR =1.36 (1.05-1.75)
[3] Due to small numbers in one or both cohorts, the exact numbers cannot be shown.

Table A2. The RR of PPAT according to the demographic factors, pregnancy-related and obstetrical events

	No of births	Of which with PPAT	RR (CI 95 %)	aRR (CI 95%)[1]
**Maternal age**				
≤ 24	4,803	26	1.48 (0.98-2.24)	1.22 (0.78-1.91)
≥ 25	52,436	183	1 (reference)	1 (reference)
**Parity**				
1	29,957	122	1.33 (1.01-1.76)	1.26 (0.94-1.70)
≥2	26,881	87	1 (reference)	1 (reference)
**Highest attained education**				
≤ 13 years of schooling	17,499	78	1.45 (1.07-1.96)	1.35 (0.97-1.89)
>13 years of schooling	34,619	102	1 (reference)	1 (reference)
**Preterm**[2]				
No	53,939	137	1 (reference)	1 (reference)
Yes	2,723	12	1.64 (0.97-2.77)	1.59 (0.94-2.70)
**Low birth weight (singletons)**				
No	53,649	>193[3]	1 (reference)	1 (reference)
Yes	1,758	<10[3]	1.10 (0.52-2.33)	1.06 (0.50-2.25)
**C-section**				
No	45,922	161	1 (reference)	1 (reference)
Yes	10,749	48	1.28 (0.93-1.77)	1.30 (0.94-1.80)
**Twins**				
No	56,272	203	1 (reference)	1 (reference)
Yes	967	6	2.43 (1.08-5.48)[4]	2.58 (1.14-5.82)[4]
**Gestational hypertension**				
No	56,039	>204[3]	1 (reference)	1 (reference)
Yes	1,200	<5[3]	0.34 (0.05-2.44)[4]	0.35 (0.05-2.47)[4]
**Preeclampsia**				
No	55,612	>196[3]	1 (reference)	1 (reference)
Yes	1,627	<13[3]	1.71 (0.88-3.34)	1.64 (0.84-3.20)
**Threatened abortion**				
No	56,169	>199[3]	1 (reference)	1 (reference)
Yes	1,070	<10[3]	2.17 (1.07-4.40)	2.15 (1.06-4.35)
**Hyperemesis gravidarum**				
No	56,343	>199[3]	1 (reference)	1 (reference)
Yes	896	<10[3]	2.15 (1.02-4.56)	2.10 (0.99-4.45)
**Gestational diabetes**				
No	55,548	>196[3]	1 (reference)	1 (reference)
Yes	1,691	<13[3]	1.43 (0.73-2.79)	1.47 (0.75-2.88)
**Maternal care for known or suspected fetal problem**				
No	52,414	193	1 (reference)	1 (reference)
Yes	4,825	16	0.93 (0.56-1.55)	0.93 (0.56-1.55)
**Fetal stress**				
No	45,833	179	1 (reference)	1 (reference)
Yes	11,406	30	0.72 (0.49-1.07)	0.65 (0.44-0.98)
**Perineal laceration**				
No	29,411	115	1 (reference)	1 (reference)
Yes	27,828	94	0.87 (0.66-1.14)	0.82 (0.62-1.09)
**Postpartum haemorrhage**				
No	43,945	153	1 (reference)	1 (reference)
Yes	13,294	56	1.22 (0.90-1.66)	1.20 (0.89-1.64)
**Puerperal sepsis**				
No	56,904	>199[3]	1 (reference)	1 (reference)
Yes	335	<10[3]	1.85 (0.46-7.43)	1.79 (0.45-7.20)

[1]Adjusted for maternal age, parity, and highest attained education.

[2] We repeated the analysis including only singletons and found RR=1.52 (0.85-2.73) and aRR=1.69 (0.89-3.21).

[3] Due to small numbers in one or both cohorts, the exact numbers cannot be shown.

[4] The estimate is based only on data from the Danish Health Visitors' Child Health Database, because of no events in the Copenhagen cohort.

Figure A. Association between the RR of PPAT and the RR of EPDS≥13 for demographic factors and pregnancy-related and obstetrical events. Based on crude RR's.

If the two measures have exactly the same risk factor pattern, the points would be on the solid line. We adjusted for maternal age, parity, and highest attained education. Young age: Maternal age ≤24 years, Primiparous: Parity 1, Short education: ≤13 years of schooling, C-section, FS: Fetal stress, GH: Gestational hypertension, GDM: Gestational diabetes, HG: Hyperemesis gravidarum, Low BW: Low birth weight, MC: Maternal care for known or suspected fetal problems, PE: Preeclampsia, PL: Perineal laceration, PPH: Postpartum haemorrhage, Preterm, PS: Puerperal sepsis, TAB: Threatened abortion, and Twins

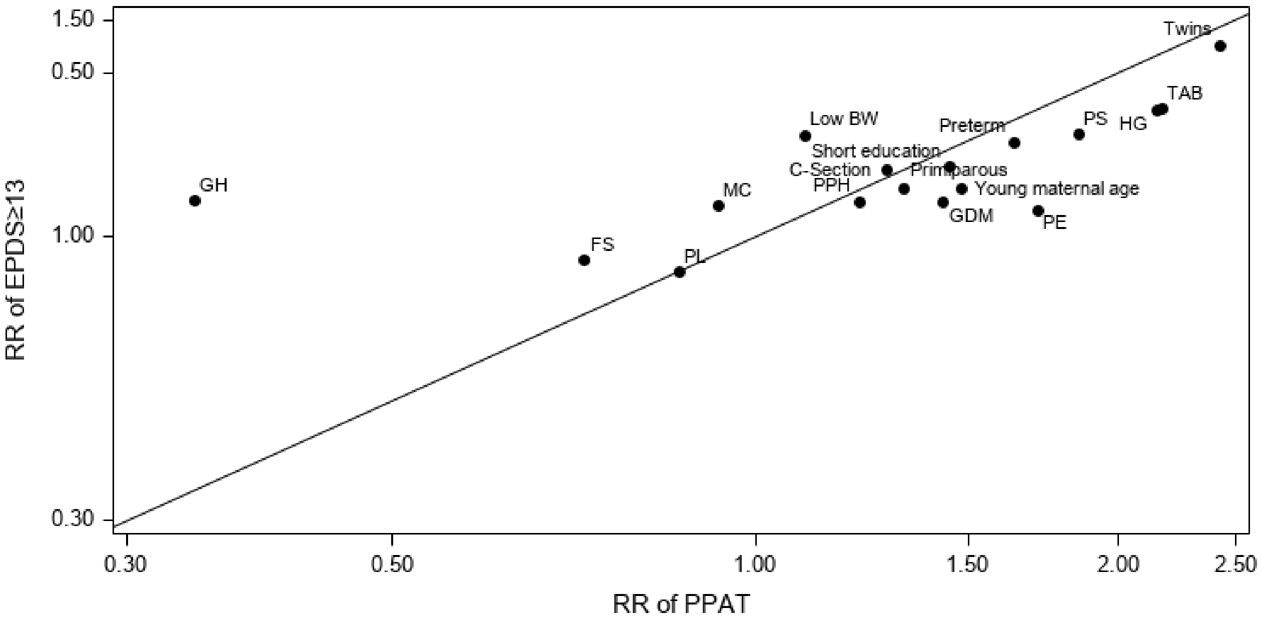

Supplement: Supplementary file 1 — Additional file 1. [file 12888_2022_3836_MOESM1_ESM.pdf]
